# Cognitive genomics: Linking genes to behavior in the human brain

**DOI:** 10.1162/netn_a_00003

**Published:** 2017-02-01

**Authors:** Genevieve Konopka

**Affiliations:** Department of Neuroscience, UT Southwestern Medical Center, Dallas, TX 75390-9111, USA.

## Abstract

Correlations of genetic variation in DNA with functional brain activity have already provided a starting point for delving into human cognitive mechanisms. However, these analyses do not provide the specific genes driving the associations, which are complicated by intergenic localization as well as tissue-specific epigenetics and expression. The use of brain-derived expression datasets could build upon the foundation of these initial genetic insights and yield genes and molecular pathways for testing new hypotheses regarding the molecular bases of human brain development, cognition, and disease. Thus, coupling these human brain gene expression data with measurements of brain activity may provide genes with critical roles in brain function. However, these brain gene expression datasets have their own set of caveats, most notably a reliance on postmortem tissue. In this perspective, I summarize and examine the progress that has been made in this realm to date, and discuss the various frontiers remaining, such as the inclusion of cell-type-specific information, additional physiological measurements, and genomic data from patient cohorts.

## INTRODUCTION

Progress in understanding the inner workings of the brain has come a long way from the preneuroscience era of phrenology, when we were limited to conjectures about human behavior based on the shape of the skull. Over the past quarter century, technological breakthroughs have given us the ability to noninvasively peer into the operations of the human brain during behavior, by means of a host of imaging and physiological techniques. Functional imaging has provided elegant maps of human activity at rest, as well as during any number of cognitive tasks. By coupling these results with neuroanatomical and structural imaging, function and structure can be married to identify brain regions that work in concert to execute specific functions. Furthermore, when such approaches are carried out in patients with neuropsychiatric disorders, the regional brain activity relevant to cognitive phenotypes can be uncovered.

## Genetic Contributions to Cognition

Determining the relative contribution of genes to cognition has been a longstanding interest in the field of genetic research. Recent inquiries have focused on unlocking the genetic and molecular mechanisms underlying human brain activity (see the discussion and references in Medland, Jahanshad, Neale, & Thompson, 2014, and Thompson, Ge, Glahn, Jahanshad, & Nichols, 2013). Key insights have been made, such as the heritability of functional brain networks (Fornito et al., 2011; Fu et al., 2015; Glahn et al., 2010; Yang et al., 2016) and the correlation of genetic variation in altered functional connectivity in specific diseases or phenotypes (see the references in Gaiteri, Mostafavi, Honey, De Jager, & Bennett, 2016; Hernandez, Rudie, Green, Bookheimer, & Dapretto, 2015). As such, these advances could have profound implications for how we diagnose and treat such disorders (see the discussion and references in Matthews & Hampshire, 2016). Furthermore, genome-wide association studies have identified specific genomic loci that are significantly associated with subcortical brain structures (Hibar et al., 2015); with educational attainment as a proxy for cognition in general (Okbay, Beauchamp, et al., 2016); with personality traits such as subjective well-being, depressive symptoms, and neuroticism (Okbay, Baselmans, et al., 2016); and with cognitive disorders such as schizophrenia (Schizophrenia Working Group of the Psychiatric Genomics Consortium, 2014). These molecular and genetic insights provide a baseline for ultimately pinpointing drug targets in a number of cognitive disorders, as well as deepening our understanding of both the developmental and evolutionary origins of human cognition. Thus, further investigations into the molecular mechanisms underlying human brain activity are needed to bridge the gap between genes and behavior.

## Quantifying Gene Expression in the Human Brain

The genome revolution, followed rapidly by implementation of the high-throughput technologies of microarrays and next-generation sequencing, has permitted investigations of human brain gene expression in a spatiotemporal manner, by quantifying RNA amounts at a genome-wide level (e.g., Kang et al., 2011). The analysis of gene transcription across the entire human brain allows for distinguishing the genes expressed in specific brain regions during a given developmental time period, and thus results in a quantitative measurement of gene expression levels. These datasets are different from the genetic associations mentioned above, in which changes at the DNA level are identified. Such genetic variation might be within regions of DNA of unknown functional significance (e.g., do the variants affect gene expression?) and might also interact with unknown epigenetic markers in a tissue-specific manner, leading to further ambiguity about the resultant gene expression. Surveying the vast transcriptional landscape of the developing and adult human brain has been facilitated by the work of the Allen Institute for Brain Science in collaboration with a number of academic groups, to develop several reference gene expression atlases of the human brain, by using a combination of in situ hybridization, microarrays, and RNA sequencing (RNA-seq) throughout the human lifespan (Hawrylycz et al., 2015; Hawrylycz et al., 2012; Miller et al., 2014; Zeng et al., 2012; see Box 1). One of the caveats to these assessments of human brain gene expression is that they are naturally limited to postmortem tissue. Although careful statistical analyses take into consideration experimental covariates such as postmortem interval and RNA quality, there is always the possibility that patterns of gene expression in behaving individuals cannot be fully recapitulated in postmortem tissue. Nevertheless, these assessments provide critical insights into human brain gene expression patterns, based on developmental stage (Kang et al., 2011; Miller et al., 2014), gender (Kang et al., 2011), hemispheric lateralization (or lack thereof; Hawrylycz et al., 2012; Johnson et al., 2009; Pletikos et al., 2014), and human-specific evolution (Bakken et al., 2016; Bernard et al., 2012). These observations can then be compared with disease-relevant datasets. For example, genetic data from autism spectrum disorder (ASD) patients were integrated with the BrainSpan gene expression dataset (www.brainspan.org) to identify ASD-relevant coexpression networks (Parikshak et al., 2013; Willsey et al., 2013). Furthermore, genomic profiling of disease tissue itself can be insightful, as has been the case for ASD, where such profiling has identified differentially expressed networks of mRNAs and microRNAs in ASD brains as compared to matched controls (Voineagu et al., 2011; Y. E. Wu, Parikshak, Belgard, & Geschwind, 2016). Together, these studies of human brain gene expression have facilitated the prioritization of specific genes and molecular pathways for further in-depth analyses. However, such follow-up studies are largely limited to animal models, and there appears to be a large divide between what can be observed at the gene expression level in brain tissue and at the behavioral level in humans.

**Box 1. Descriptions of Gene Expression Detection Methods**

*In situ hybridization* is carried out by hybridizing gene-specific RNA probes to tissue samples. This method provides spatial resolution of the mRNA expression of individual genes. High-quality tissue specimens and highly specific probes to each gene are required for accurate detection. A major advantage of in situ hybridization is the ability to couple it with immunohistochemistry to make mRNA and protein correlations. However, the quantification of in situ hybridization is challenging, depending on the method used. The greatest advantages of in situ hybridization over the other technologies discussed below are its spatial resolution and ability to detect expression from small amounts of tissue.

*Microarrays* for gene expression rely on hybridization of an RNA sample to a chip spotted with predetermined oligonucleotide probes. Although recent microarrays can provide full genome coverage and can detect, for example, small RNAs, the technology is still not completely unbiased. For one thing, novel gene transcripts and unannotated regions of a genome will not be detected or quantified using microarrays. Moreover, microarrays are less useful for querying expression in species for which specific microarrays are not available, nor will microarrays provide cellular-resolution expression information. However, well-established algorithms exist for analyzing microarray data, and their results are highly consistent when comparing many samples on the same type of microarray. The processing and analysis of microarray data is also less costly in terms of time, money, and computational power needed.

*RNA sequencing*, or *RNA-seq*, uses next-generation sequencing technology to quantify the expression of all expressed genes in an RNA sample in an unbiased manner, without any a priori information about the sample. Expression information is limited by howthe RNA sample is processed—that is, is all of the RNA being processed, or are polyadenylated transcripts selected? Expression information can also be limited by the length of the sequencing read (e.g., 50 vs. 150 base pairs, or single- vs. paired-end reads) and the amount of sequencing depth carried out. Such parameters can limit the ability to make informed analysis of features such as the expression of noncoding RNAs or RNA splicing. In addition, there is no agreedupon method for analyzing RNA-seq data. However, all of these analyses and more can be carried out, given the appropriate sample preparation, sequencing method, and analytical pipeline. RNA-seq alone will not provide cellular-level resolution unless it is coupled with other technologies, such as microfluidics or flow cytometry (e.g., single-cell RNA-seq). The advantages of RNA-seq include the unbiased detection of expression, which makes the technology compatible with any species of interest and any type of RNA, and a larger dynamic range of detection thanwithmicroarrays (i.e., it is easier to detect low-abundance expression).

Descriptions of Gene Expression Detection Methods *In situ hybridization* is carried out by hybridizing gene-specific RNA probes to tissue samples. This method provides spatial resolution of the mRNA expression of individual genes. High-quality tissue specimens and highly specific probes to each gene are required for accurate detection. A major advantage of in situ hybridization is the ability to couple it with immunohistochemistry to make mRNA and protein correlations. However, the quantification of in situ hybridization is challenging, depending on the method used. The greatest advantages of in situ hybridization over the other technologies discussed below are its spatial resolution and ability to detect expression from small amounts of tissue.

*Microarrays* for gene expression rely on hybridization of an RNA sample to a chip spotted with predetermined oligonucleotide probes. Although recent microarrays can provide full genome coverage and can detect, for example, small RNAs, the technology is still not completely unbiased. For one thing, novel gene transcripts and unannotated regions of a genome will not be detected or quantified using microarrays. Moreover, microarrays are less useful for querying expression in species for which specific microarrays are not available, nor will microarrays provide cellular-resolution expression information. However, well-established algorithms exist for analyzing microarray data, and their results are highly consistent when comparing many samples on the same type of microarray. The processing and analysis of microarray data is also less costly in terms of time, money, and computational power needed.

*RNA sequencing*, or *RNA-seq*, uses next-generation sequencing technology to quantify the expression of all expressed genes in an RNA sample in an unbiased manner, without any a priori information about the sample. Expression information is limited by how the RNA sample is processed—that is, is all of the RNA being processed, or are polyadenylated transcripts selected? Expression information can also be limited by the length of the sequencing read (e.g., 50 vs. 150 base pairs, or single- vs. paired-end reads) and the amount of sequencing depth carried out. Such parameters can limit the ability to make informed analysis of features such as the expression of noncoding RNAs or RNA splicing. In addition, there is no agreed-upon method for analyzing RNA-seq data. However, all of these analyses and more can be carried out, given the appropriate sample preparation, sequencing method, and analytical pipeline. RNA-seq alone will not provide cellular-level resolution unless it is coupled with other technologies, such as microfluidics or flow cytometry (e.g., single-cell RNA-seq). The advantages of RNA-seq include the unbiased detection of expression, which makes the technology compatible with any species of interest and any type of RNA, and a larger dynamic range of detection than with microarrays (i.e., it is easier to detect low-abundance expression).

## Correlations of Human Brain Gene Expression With Functional Imaging Data

**Figure 1.**
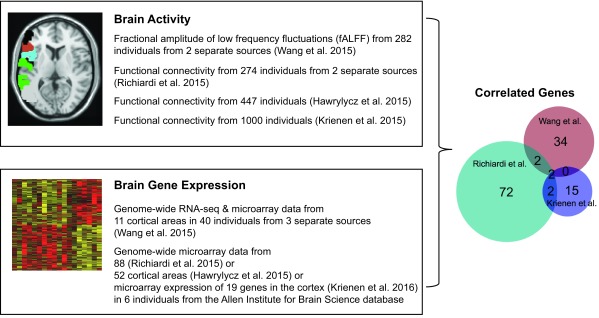
Integrating functional brain activity and gene expression in the human brain has resulted in the identification of a small group of genes that are likely important for resting-state functional networks.

Bridging the divide between genes and behavior in human brains has entered a new chapter with the first studies to compare brain gene expression and functional imaging datasets from humans (Fig. 1) (Hawrylycz et al., 2015; Krienen, Yeo, Ge, Buckner, & Sherwood, 2016; Richiardi et al., 2015; G. Z. Wang et al., 2015). These studies utilized functional magnetic resonance imaging (fMRI) data obtained during the resting state. It remains to be determined whether gene expression data from postmortem tissues are more related to brain activity during the resting state or better reflect an “acute” active state resulting from a specific function or task. Since detailed behavioral data from brain donors are unavailable, resting-state correlations are a reasonable starting point. In addition, transcriptional responses to stimuli are on the order of minutes, whereas postmortem times are typically on the order of hours, again supporting relevance to the resting state. Finally, all of these studies focused their comparisons on the neocortex; one reason for this is the relative similarity in gene expression across cortical areas, relative to the large gene expression differences when comparing cortical to noncortical areas (Hawrylycz et al., 2015), and the other reason is the availability of independent cortical expression datasets.

In the study by Richiardi et al. (2015), four well-characterized functional networks from 15 subjects were compared to microarray data from high-resolution gene expression profiling of six postmortem brains that was carried out by the Allen Brain Institute. This comparison identified ∼78 genes significantly correlated with the functional networks. Common polymorphisms in these genes were further found to correlate with functional networks in the larger (∼259 subjects) IMAGEN cohort, for whom both single-nucleotide polymorphism and fMRI data were available (Schumann et al., 2010). Finally, Richiardi et al. further validated the gene list in mouse orthologs, showing that the expression of these genes correlated with mouse brain connectivity. The correlated gene list was enriched for neuron-specific genes and for genes encoding ion channels, as well as for genes associated with Alzheimer’s disease and schizophrenia. These results were consistent with a study by Hawrylycz et al. (2015) that used the same gene expression dataset, along with independent functional connectivity MRI data from 447 subjects that are part of the Human Connectome Project (Van Essen et al., 2013). In the Hawrylycz et al. (2015) study, the authors set out to determine the genes with the most consistent pattern of gene expression across human brains. Using the genes with the most consistent differential expression across the cortex, they found that the same genes identified in Richiardi et al. (2015) were among the genes with the greatest correlations of expression with functional connectivity.

In G. Z. Wang et al. (2015), RNA-seq and microarray data from three independent datasets were used, containing samples from approximately 40 individuals. Each of these three gene expression studies was compared to two independent fMRI datasets, with 84 and 198 subjects, and the consensus of the correlations within the default-mode network was reported. In this study, a limited number of cortical areas were included (five to ten), due to sample availability. Nevertheless, 38 genes were identified that correlated significantly with the default-mode network. These genes were also enriched for neuron-specific genes, genes encoding ion channels, and genes implicated in ASD. In particular, genes relevant to interneuron identity were suggested to play a potentially key role in orchestrating the brain activity assessed by fMRI.

One of the challenges of interpreting the results from these first studies to correlate human-brain gene expression and fMRI signals is that the correlations across regions might be biased toward differences in cell-type proportions in these regions. Hence, the identified enrichment of neuron-specific genes could be an artifact of the relative variation in neuronal proportions in these cortical regions. This is further exemplified by the finding that human brain gene expression is most conserved with respect to mouse brain gene expression among neuron-relevant genes (Hawrylycz et al., 2015), and the correlations between gene expression and functional connectivity are conserved between human and mouse brains (Richiardi et al., 2015). The distinctions in areal and cell-type-specific expression patterns driving the correlations with activity should be addressed with improved technology for single-cell expression profiling and higher-resolution imaging, as will be discussed below. One additional challenge in interpreting these findings is that there is no manner in which to assign causation to the correlations. In other words, there is no way to determine whether the gene expression patterns are a result of brain activity, or whether the brain activity is somehow driven by the gene expression patterns. One way to assess this would be to measure gene expression in vivo before, during, and after a task, something that is clearly not possible in humans. However, there has been remarkable progress in following the translation of single mRNAs in live cells, including neurons, as well as in living animals such as Drosophila (Halstead et al., 2015; C. Wang, Han, Zhou, & Zhuang, 2016; B. Wu, Eliscovich, Yoon, & Singer, 2016; Yan, Hoek, Vale, & Tanenbaum, 2016). Therefore, such technology might one day be available for the study of humans.

Because the gene expression data discussed here derive from postmortem sources, these studies require the use of separate cohorts from MRI studies for comparison. In other words, the gene expression data were not derived from tissue from the subjects who underwent fMRI. In addition, there was no overlap between the imaging and gene expression cohorts across the Richiardi et al. (2015) and the G. Z. Wang et al. (2015) studies, nor were the methods or networks analyzed in the same way. For example, Richiardi et al. examined correlations across brain regions in four networks (dorsal default-mode, salience, sensorimotor, and visuospatial), whereas G. Z. Wang et al. focused on the default-mode network and an indicator of activity, fractional amplitude of low-frequency fluctuations (fALFF), from within each individual region, followed by correlations across the regions. These differences make it all the more remarkable that the results displayed statistically significant overlap in the genes identified (G. Z. Wang et al., 2015). For example, *NECAB2*, *NEFH*, *SCN1B*, and *SYT2*were identified in both the Richiardi et al. and G. Z. Wang et al. studies. Such overlaps suggest that even when using different cohorts and different methods, the expression of genes underlying brain activity at rest can be identified. Perhaps even more remarkable were the results of a subsequent study by Krienen et al. (2016) that focused on 19 genes enriched in human supragranular layers and that identified specific corticocortical connectivity networks correlated with these genes using functional connectivity MRI measurements. Both *NEFH* and *SYT2*are among these 19 genes. Although the human gene expression data in the Krienen et al. study are the same as in the Richiardi et al. and Hawrylycz et al. studies, the imaging dataset was derived from an independent cohort of 1,000 subjects from the Brain Genomics Superstruct Project (Holmes et al., 2015). Thus, these overlaps in genes suggest intriguing hypotheses to test regarding the roles of these specific genes in directing human-specific corticocortical connections. For example, are there genetic polymorphisms or differential expression patterns of these two genes in patients with either abnormal connectivity patterns, as assessed by imaging, or abnormal cognitive phenotypes or disorders? There are already examples of altered NEFH levels in brain tissue from individuals with schizophrenia (Pinacho et al., 2016) or alcoholism (Iwamoto et al., 2004); altered levels of *SYT2* mRNA in schizophrenia (McMeekin et al., 2016); and association of *SYT2* with both attention-deficit/hyperactivity disorder (Sánchez-Mora et al., 2013) and cocaine dependence (Fernàndez-Castillo et al., 2012). Whether these changes and variants can be linked to neuroanatomical alterations remains to be determined.

Together, these studies build upon previous work that has supported a genetic basis for functional connectivity and brain activity, showing evidence for mRNA expression correlations with these measurements. The genes identified are synaptic genes enriched in neurons; however, it is likely that this list of genes is not exhaustive, and as further detailed genomic profiling is carried out, additional refined lists will be uncovered. The differences among the genes identified raise important considerations. G. Z. Wang et al. (2015) only used data from male subjects, due to sample availability, in one of the gene expression studies. It is unlikely that gene list differences arose from such sample constraints, but as greater numbers of cortical RNA-seq datasets become available, those differences can be tested. In addition, as was previously mentioned, G. Z. Wang et al. used a region-of-interest approach to calculate activity, while the other studies employed functional connectivity. Cognition can be studied at a network level, and these networks span the gamut from those quantitated with fMRI, down to those assessed by RNA sequencing (Petersen & Sporns, 2015). However, before these initial studies using human datasets were carried out, it was not known whether gene expression patterns would fall in line with the networks observed using functional connectivity approaches. The gene expression data are processed from each region independently of the others, and the data can be collated from different individuals within the same brain regions with minimal variation (Hawrylycz et al., 2012). In addition, the inability to directly manipulate gene expression or brain regional activity in humans makes testing the contribution of the observed gene list to cognitive function challenging. For example, as was already mentioned, there is no way to test causation in humans: that is, whether the level of expression of a particular gene might result in altered brain activity, or whether altered brain activity results in a change in gene expression. However, such experiments could be carried out in animal models if the same correlations between expression and activity were valid.

## Comparisons With Nonhuman Brain Expression and Activity

How, then, do these results in humans compare to what was already known in nonhuman brains? Such comparison would open up the possibility of translating the observations in humans into model systems. Initial work in worms provided direct evidence for gene expression signatures indicating neuronal connectivity (Kaufman, Dror, Meilijson, & Ruppin, 2006). Subsequent studies in rodents have supported the idea of brain gene expression correlating with structural connectivity (French & Pavlidis, 2011; Ji, Fakhry, & Deng, 2014; Wolf, Goldberg, Manor, Sharan, & Ruppin, 2011). Interestingly, the gene ontology of these correlated genes suggests a role for neuronal projection and guidance molecules in connectivity, as might be predicted. In another study, the genes correlated with network hub connectivity were identified as those involved in energy metabolism (e.g., oxidative synthesis and ATP metabolism; Fulcher & Fornito, 2016), highlighting genes of interest for studies of brain disorders featuring both metabolic deficiencies and abnormal connectivity, such as Alzheimer’s disease. In addition, a significant number of ASD-relevant genes were among the structurally correlated genes (French & Pavlidis, 2011) as in the findings of G. Z. Wang et al. (2015). Moreover, follow-up work using human samples suggested that not only are there correlations between gene expression and structural connectivity, but that gene expression might be one of the driving forces behind the observed connectivity (Goel, Kuceyeski, LoCastro, & Raj, 2014), and therefore a more causative correlation between these features might be discernible.

From a global gene expression perspective, the recent detailed profiling of the human brain transcriptome has allowed for extensive comparisons with both the mouse and rhesus macaque brain transcriptomes (Bakken et al., 2016; Hawrylycz et al., 2012). These comparisons have uncovered highly correlated expression in similar regions of the human and mouse brains, particularly when the genes were more related to neuronal expression patterns (Hawrylycz et al., 2015). This is in line with data from Richiardi et al. (2015) that demonstrated similar correlations of mouse brain gene expression and mouse brain connectivity when the same gene list identified in the human brain was applied to the mouse. Of note, some genes vary in their brain expression patterns between species (Hawrylycz et al., 2015), and it would be interesting to examine whether these particular genes demonstrate differential correlations with human brain activity measurements in patient populations with cognitive disorders. Such human-relevant expression differences become even more notable when rhesus macaque is included in the comparisons, since this allows for comparison with a more closely related primate species, rather than the mouse. The study by Bakken et al. (2016) compared human, rhesus macaque, rat, and mouse brain expression, including developmental expression data as well as some cortical-layer data. While the authors confirmed and further delineated genes with conserved patterns of expression in mammalian brain, they also identified a subset of genes that demonstrate human-specific expression patterns across cortical development. Again, examining whether the expression pattern of these genes over development show differential correlations with developmental functional brain activity could be relevant to understanding cognitive disorders. Together, these studies suggest that conserved gene expression across brain regions underlies many features of mammalian brain structure and activity, and they also prioritize specific genes for detailed study in animal disease models.

## Future Directions

In the last few years, there has been significant progress into elucidating the molecular mechanisms of human brain functional networks. We know that there is a genetic, heritable component to these brain networks, as well as patterns of gene expression that may direct them. We have also learned that these genes and their expression may be tied to the human brain’s evolution and cognitive disorders. What, then, are the next frontiers for delving deeper into these mechanisms?

In recent years, efforts have been made to standardize experimental approaches in order to permit the collation and comparison of datasets across studies. This has been particularly noteworthy among both imaging and genetic consortia. Recently, these collaborative efforts on the imaging front have expanded to include imaging of specific cognitive disorders. For example, the ABIDE consortium has provided novel insights into ASD (Di Martino et al., 2014), and the IMAGEN Consortium (Schumann et al., 2010) has combined genetics and imaging to study a variety of phenotypes relevant to psychiatric disorders. The Human Connectome Project (Glasser, Coalson, et al., 2016; Glasser, Smith, et al., 2016) has given these and all other imaging studies a new baseline from which to derive deviations from the norm. In the field of neurogenetics, this collaborative approach has also been critical for tackling assessments of genetic associations with disease. Recent large-scale efforts to examine schizophrenia (Schizophrenia Working Group of the Psychiatric Genomics, 2014) and depression (CONVERGE Consortium, 2015) have required the inclusion of tens of thousands of samples just to scratch the surface of the common genetic features of these disorders. The deposition of raw gene expression data from human brain tissue into repositories such as NCBI GEO, alongside the efforts of the Allen Brain Institute (http://human.brain-map.org (Hawrylycz et al., 2015; Hawrylycz et al., 2012) and the BrainSpan Consortium (www.brainspan.org), has further permitted detailed analyses and hypothesis testing by many research groups. The fruits of all of these efforts can now be combined in a number of ways to ask whether the correlations between gene expression and brain activity differ across multiple parameters, such as endophenotypes, medication use and response, or other comorbidities when queried in patient populations. The abundance of genetic information from the increasing number of genome-wide association studies and large-scale whole-genome sequencing efforts can also be layered into these analyses, when expression data are lacking from a particular patient group, to make predictive correlations regarding whether a particular gene might be expected to have altered expression in a particular disorder.

Of course, an ideal scenario would take a longitudinal approach in which populations of patients and unaffected controls could be genotyped, phenotyped, imaged, and followed throughout the lifespan, culminating in tissue donation (see, e.g., the MyConnectome project: Poldrack et al., 2015). Such longitudinal within-subjects analyses could last longer than the average research career of individuals, but ultimately they would be quite informative for our understanding of cognitive disorders. Although transcriptional data would necessarily be collected at only one time point, the aggregation of data across many individuals might permit inferences about how gene expression is relevant to specific phenotypes. Delimited within-subjects approaches could also potentially be used with surgical patients. For example, surgical patients for drug-resistant epilepsy not only have the epileptic foci removed during surgery, but they might also require removal of the adjacent unaffected tissue to obtain access to the foci (Spencer, Spencer, Mattson, Williamson, & Novelly, 1984). Such patients who undergo preoperative imaging studies could be included in a within-subjects study to correlate brain activity or structure from the imaging study with gene expression profiling from the surgical resections. In addition, other measurements of brain activity in these patients, such as intracranial electroencephalography or single-unit recordings, could be coupled with measurements of gene expression obtained from the surgically resected tissue.

Finally, one of the most exciting advances on the gene expression front has been the adaptation of fluidics and improved next-generation sequencing library preparations to permit the assessment of genome-wide gene expression in single cells or single nuclei, with minimal signal amplification biases. These approaches have been applied to both surgical and postmortem brain tissue from humans (Darmanis et al., 2015; Johnson et al., 2015; Krishnaswami et al., 2016; Lake et al., 2016; Pollen et al., 2015). Such studies are revising the definitions of cell types in the brain, and their data have important downstream implications for how researchers can interpret the phenotypic outcomes of genetic variants in patients or animal models. While it is unlikely that genomic profiling of every cell in a human brain will be carried out in the near future, discrete assessment of particular brain regions relevant to a particular disorder, or samples derived from surgical patients who had undergone functional or physiological assessment, should be particularly revealing. For example, the study by Lake et al. (2016), in which they profiled over 3,000 nuclei across six cortical areas from a postmortem brain, provided evidence to support the idea of brain region and local heterogeneity among neuronal subtypes, thus further emphasizing the need to explore these relationships at the cellular level. The incorporation of improved parcellation maps of the human brain (Ding et al., 2016; Glasser, Coalson, et al., 2016) should also further facilitate integrating measurements of gene expression that take into account regional heterogeneity with functional measurements. For example, these new maps could direct refinements of postmortem tissue dissections for gene expression studies. It is not yet clear how to ideally integrate cellular-resolution gene expression data with similar resolutions of brain activity, should such levels be achievable in humans. For example, network approaches with cellular resolution that work in genetically modified animals or with viral injections (e.g., calcium sensors such as GCaMP) await future advances in imaging for a less-invasive approach in humans. The advent of noninvasive approaches, so-called “molecular fMRI,” in nonhuman systems may eventually translate into human studies (Bartelle, Barandov, & Jasanoff, 2016). This approach would utilize a probe (e.g., a reporter with a neuron-specific driver introduced virally via injection) that can provide a quantitative readout to detect changes in gene expression at a cellular level when it is coupled with MRI. However, in the meantime, one could imagine that less high-throughput comparisons using electrophysiological methods in cultured human neurons or brain organoids could still be illuminating. Because strong correlations with direct physiological stimulations and blood-oxygen-level dependent signals, and between single-cell transcriptomics and electrophysiology, have been achieved in rodents (Lee et al., 2016; Tasic et al., 2016), it is possible that a connection between fMRI, molecular or physiological stimulation in vivo, and gene expression could be made in humans.

While connecting genes to behavior is an ultimate goal for many neuroscientists, the many layers of regulation intervening between DNA, the ensemble of cellular activities, and the multitude of circuit and network combinations make deciphering these connections challenging. Moreover, these relationships are further complicated by cell-specific epigenetics and gene expression, and by a greater appreciation of somatic mutations during human brain development (Lodato et al., 2015). Nevertheless, as has been evidenced by the few studies discussed here in detail, major inroads are being made by linking mRNA levels in the brain to fMRI studies in humans. Clearly, more has yet to be accomplished. However, these first steps have opened up the possibility of combining diverse human brain datasets to achieve new insights into the molecular mechanisms of cognitive functions.

## ACKNOWLEDGMENTS

I thank Maria Chahrour and the members of the Konopka lab for comments on the manuscript. The research in the Konopka lab is supported by grants from NIMH, NIDCD, the NSF, the U.T. BRAIN initiative, the Simons Foundation, and the James S. McDonnell Foundation.

## TECHNICAL TERMS

Microarrays: Technology that uses preselected oligonucleotides to quantify RNA amounts on a genome-wide basis. (See Box 1.)
In situ hybridization: Probe-based technology used to assess the spatial expression of specific RNA molecules (See Box 1.)
RNA sequencing (RNA-seq): Unbiased quantification of RNA molecules on a genome-wide basis. (See Box 1.)
Functional magnetic resonance imaging (fMRI): Noninvasive method for measuring brain activity, based on blood oxygenation levels that provide spatial and temporal resolution on the order of millimeters and seconds, respectively.
Fractional amplitude of low-frequency fluctuations (fALFF): Measurement of brain activity from fMRI datasets that compares signals derived from low frequencies (0.01–0.08 Hz) to all frequency signals.
Transcriptome: The entire set of transcripts or messenger RNAs from a particular tissue and/or organism.
Endophenotype: Phenotype(s) related to a specific disorder that are quantifiable and have a genetic component.
Electroencephalogram (EEG): Noninvasive method for measuring brain activity, on the basis of electrical activity assessed with electrodes placed on the scalp.
